# Supplementary material for: Sensitivity of quantitative diffusion MRI tractography and microstructure to anisotropic spatial sampling
Source: Magn Reson Imaging. Author manuscript; Available in PMC 2026 Jul 24. (PMC13398146; doi:10.1016/j.mri.2025.110539)
Supplement: 1 [file NIHMS2184641-supplement-1.docx]

Supplemental Material 1: Axial diffusivity and Radial Diffusivity analysis


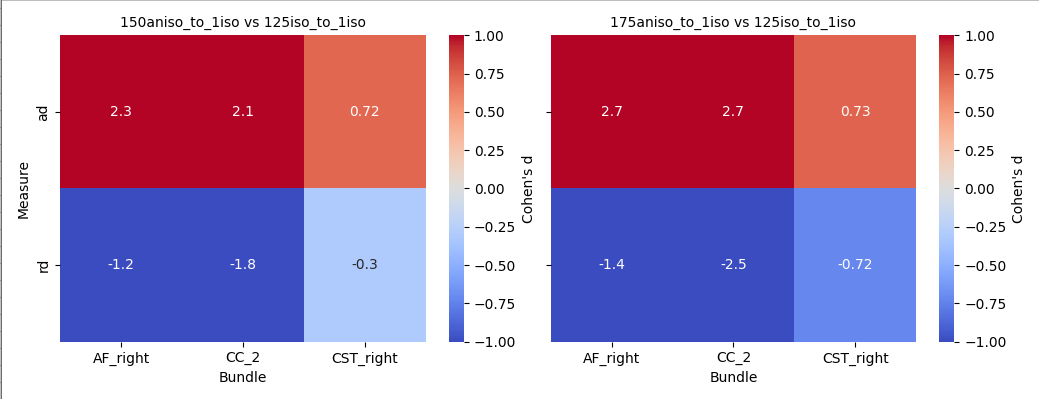


Supplemental Material 1: Effect sizes between resampled anisotropic images between axial diffusivity (AD) and radial diffusivity (RD) computed with the same protocol as FA and MD within section 2.3 and Cohen’s *d* computation consistent with section 3.1. With an increase in anisotropic smoothing from the down-sampling step, the absolute value of the effect sizes increases for both metrics.

Supplemental Material 2


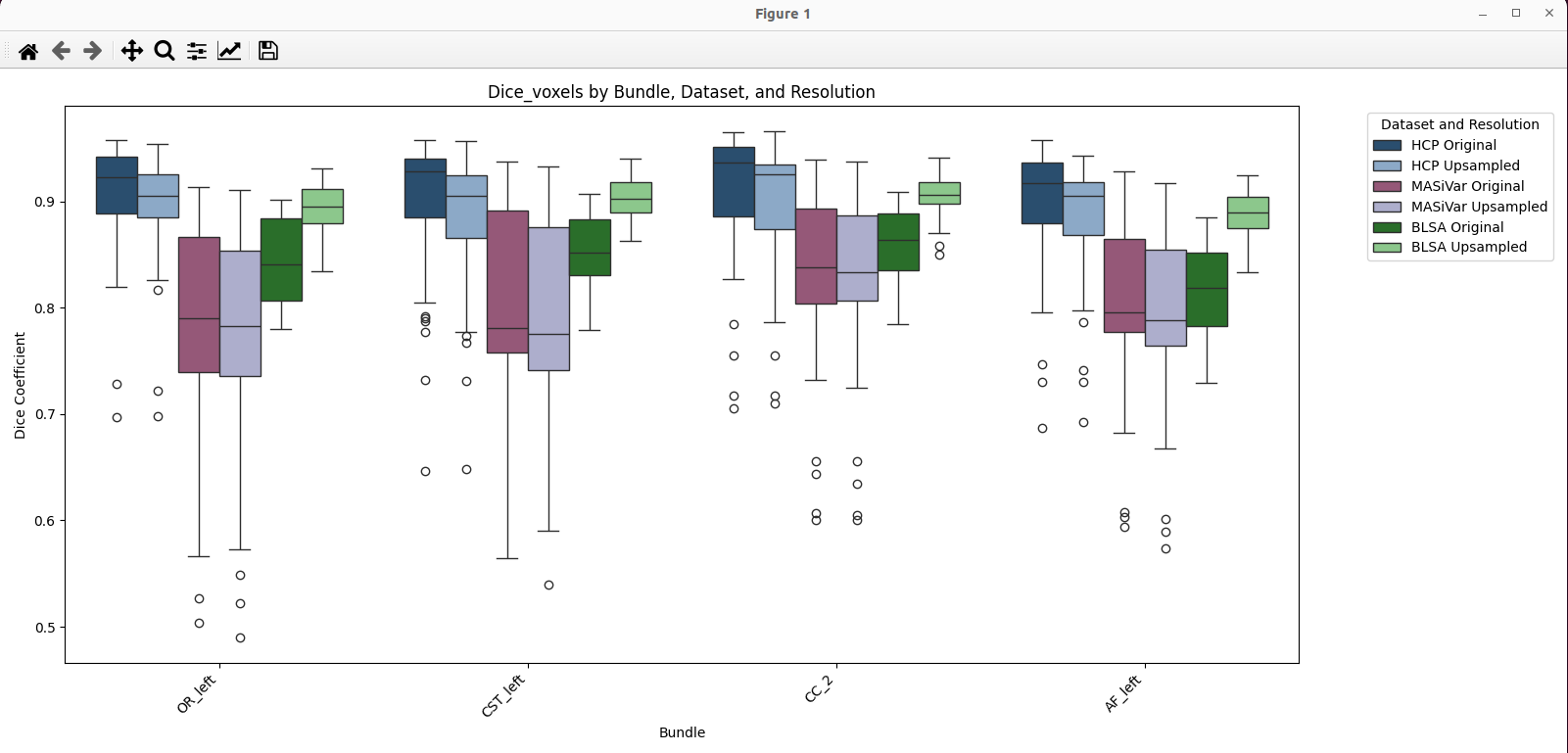


Supplemental Material 2: TractSeg’s bundle segmentation function’s scan-rescan variability across two out-of-distribution datasets, a subset of healthy aging adults from BLSA (processed with resolution 0.8125 mm x 0.8125 mm x 2.2 mm) and a pediatric subset of MASiVAR (2.14286 mm x 2.14286 mm x 2.2 mm). We use preprocessing consistent with section 2.3 at native resolution followed by resampling to 1 mm isotropic. We use TractSeg to extract bundle segmentations. Notably, resampling improves Dice voxels agreement within the aging adult cohort. Since the bundle segmentation drives tractography generation, this real-world, clinical scenario demonstrates the importance of resampling anisotropic data in preparation for TractSeg.

Supplemental Material 3: Deterministic tractography across resolutions

**
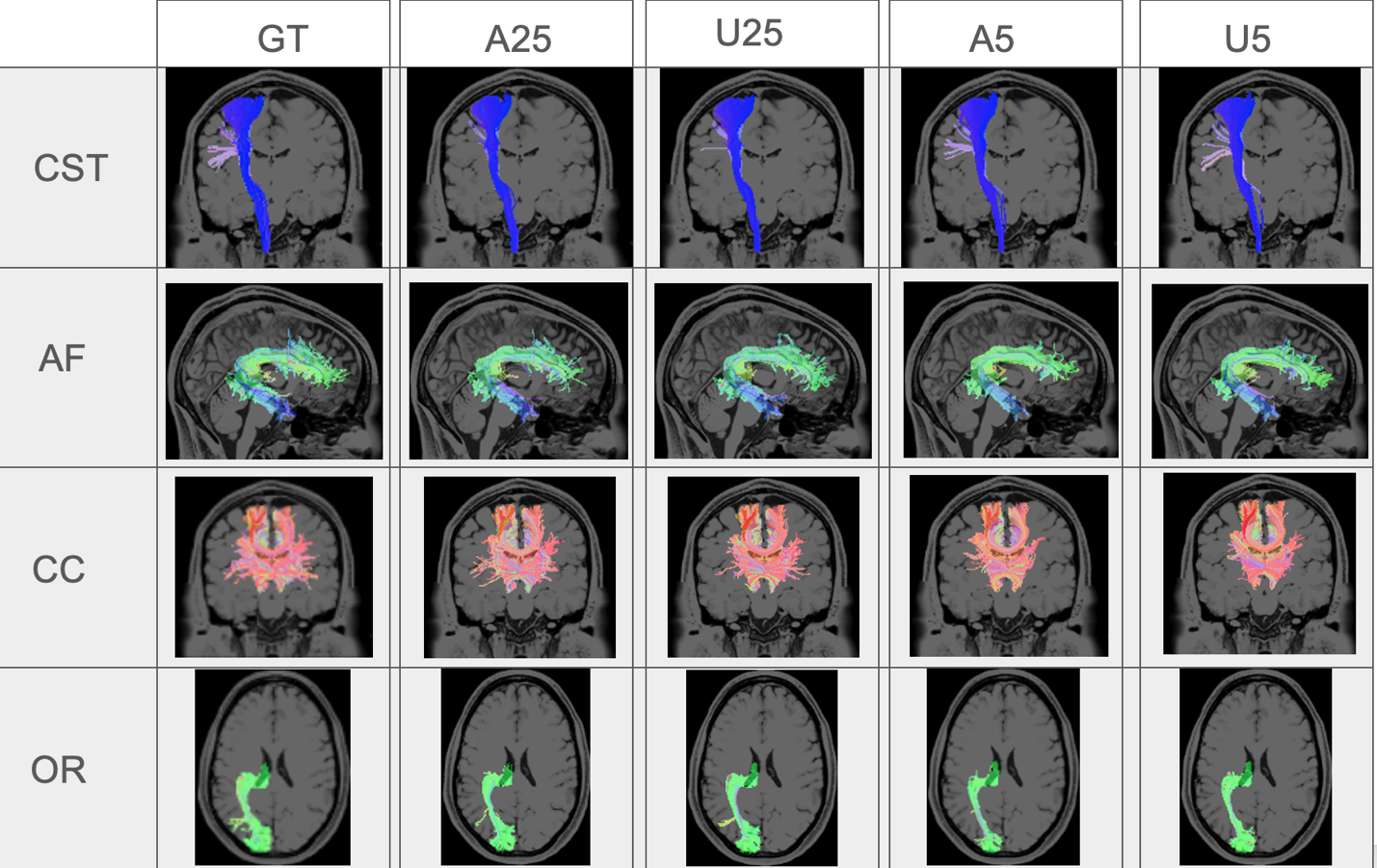
**

**
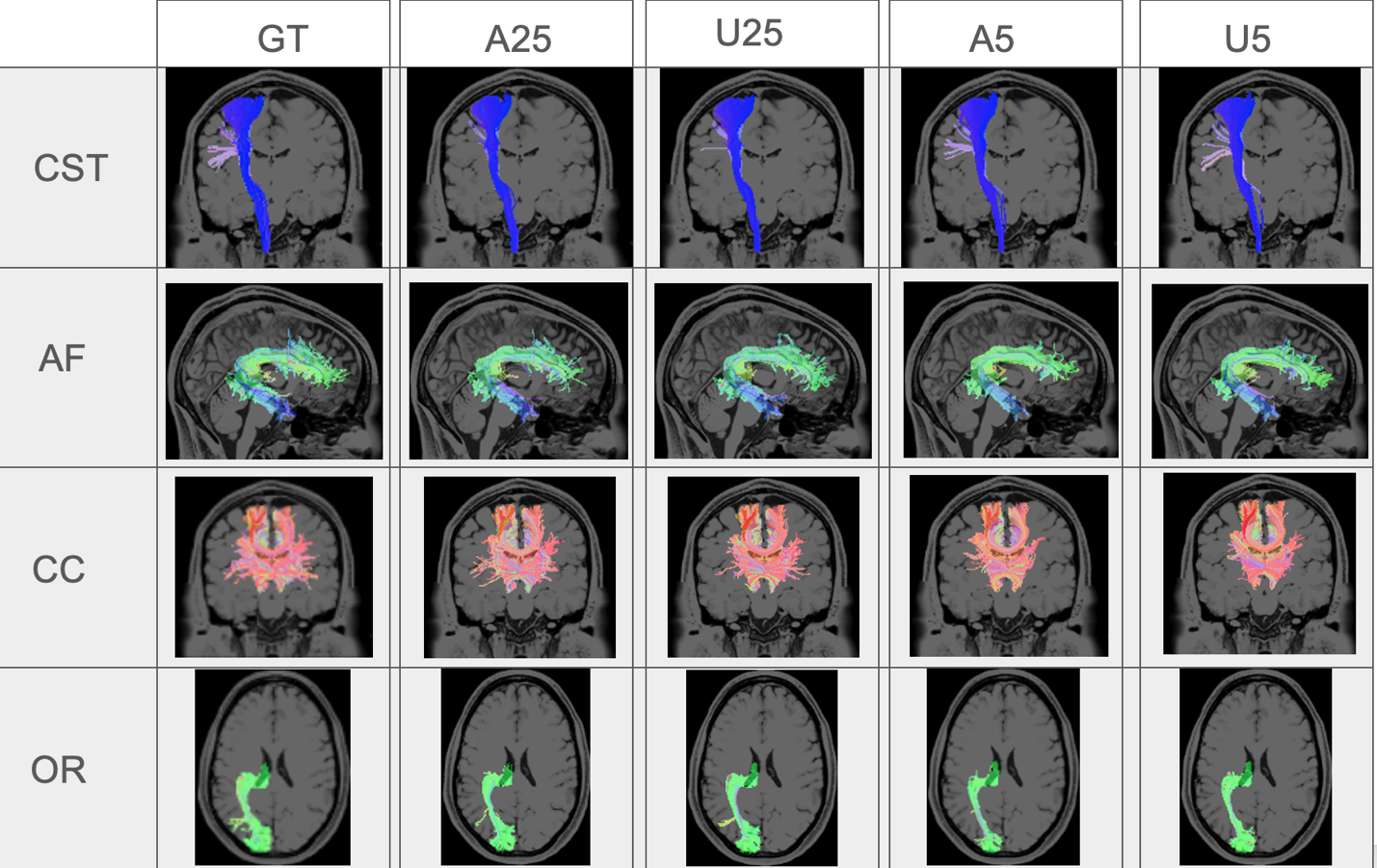
**

We evaluate the effect of spatial sampling in the FACT algorithm on a single subject with a control for bundle mask, bundle beginning and endpoints, and subject anatomy, in an ISMRM 2025 abstract, titled “Tractography based white matter tract segmentation is robust to anisotropic resolutions.” From the ground truth (GT) scan at HCP resolution (1.25 mm isotropic), we extract TractSeg segmentations and bundle beginning and endpoint and perform FACT tractography. Then, the GT is downsampled to 1.25 mm x 1.25 mm x 2.5 mm (A25) and 1.25 mm x 1.25 mm x 5 mm (A5); the anisotropic images are upsampled (U25, U5) to GT resolution following downsampling. Despite the efforts to control for variation, visible qualitative differences between the bundles appear. This suggests that spatial sampling, even with different tractography approaches, drives significant variation.

Supplemental Material 4: Elongation of the x and y axes


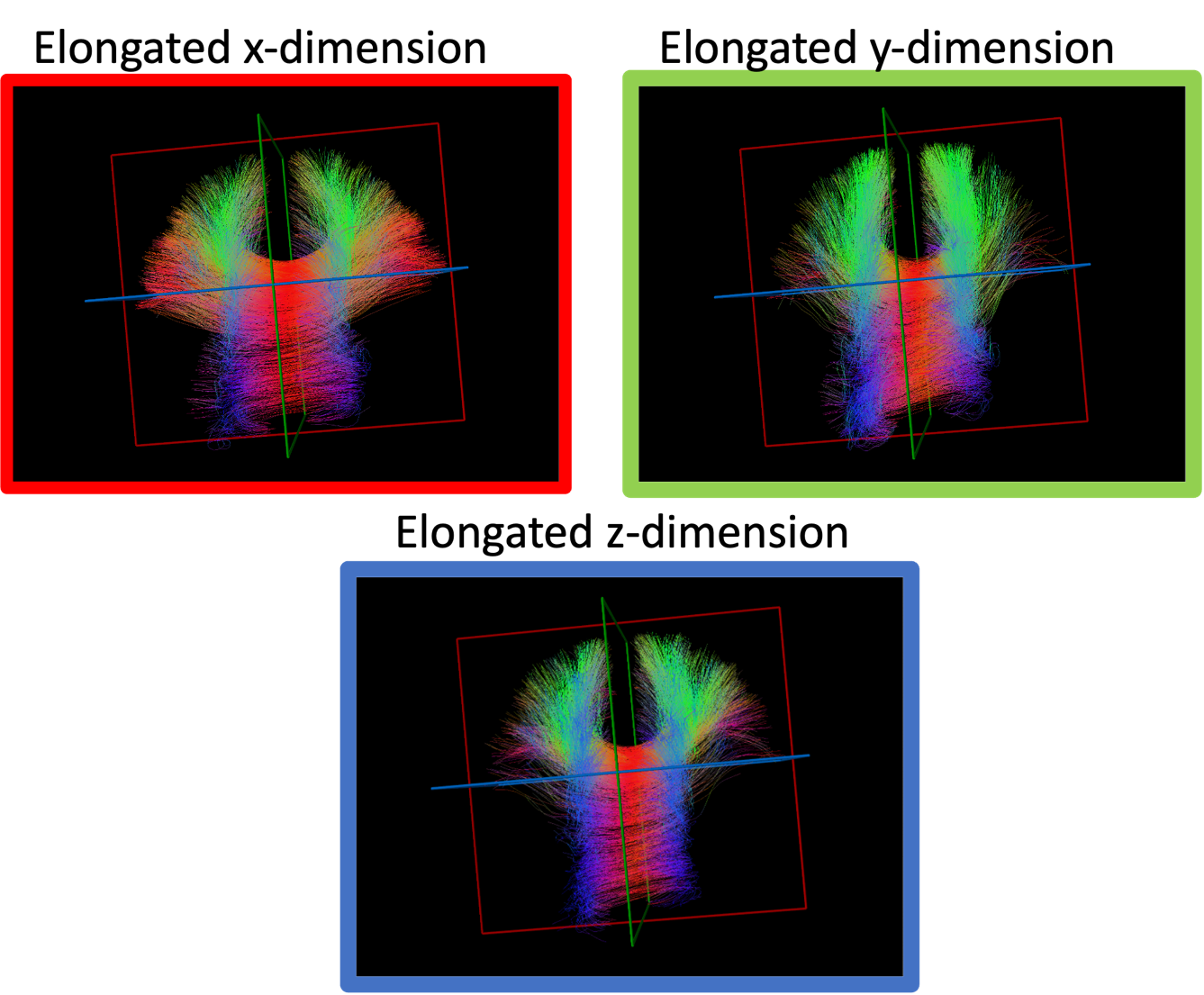


Supplemental Material 4: In a one-subject experiment to investigate the qualitative biases induced by anisotropic voxels in the CC_2, one dimension is elongated from 1.25 mm to 1.75 mm consistent with section 2.2. Overall, streamlines appear to cluster in the direction of the elongation, which suggests a bias toward the direction of anisotropy instead of a consistent representation of anatomy.
